# ZD6474 – clinical experience to date

**DOI:** 10.1038/sj.bjc.6602604

**Published:** 2005-05-31

**Authors:** J V Heymach

**Affiliations:** 1 Lowe Center for Thoracic Oncology, Dana Farber Cancer Institute, 44 Binney St, Boston, MA 02115, USA

**Keywords:** ZD6474, antitumour, VEGFR inhibitor, EGFR inhibitor, non-small-cell lung cancer

## Abstract

ZD6474 selectively targets two key pathways in tumour growth by inhibiting vascular endothelial growth factor (VEGF)-dependent tumour angiogenesis and epidermal growth factor (EGF)-dependent tumour cell proliferation and survival. Phase I clinical evaluation has shown ZD6474 to be generally well tolerated, with a pharmacokinetic profile appropriate for once-daily oral dosing. Phase II evaluation of ZD6474 at doses of 100−300 mg is ongoing in a range of patient types in single and combination regimens. These include three randomised studies of patients with non-small-cell lung cancer. In one of these trials, the efficacy of ZD6474 monotherapy is being compared with that of the EGF receptor tyrosine kinase inhibitor gefitinib (Iressa™) in previously treated patients. In the other two trials, the efficacy of ZD6474 in combination with certain standard chemotherapy regimens is being compared with that of standard chemotherapy alone: one with carboplatin and paclitaxel in previously untreated patients, and the second with docetaxel in patients who progressed after platinum-containing therapy. The advent of novel molecular-targeted agents such as ZD6474 has necessitated a re-evaluation of conventional cancer study design in order to optimise appraisal of this new generation of anticancer agents. The specific considerations of the ZD6474 clinical programme are discussed.

It is generally accepted that significant advances in cancer treatment will require the identification of novel targets critical for cancer growth and progression, and the development of strategies to inhibit these targets. Two promising targets are the vascular endothelial growth factor (VEGF) pathway, which is a key mediator of tumour angiogenesis, and the epidermal growth factor receptor (EGFR). The therapeutic value of inhibiting these targets has been demonstrated by the anti-VEGF monoclonal antibody, bevacizumab (Avastin™), the anti-EGFR monoclonal antibody cetuximab (Erbitux™) and the EGFR tyrosine kinase inhibitors erlotinib (Tarceva™) and gefitinib (Iressa™) (Fukuoka *et al*, 2003; Cunningham *et al*, 2004; Hurwitz *et al*, 2004; Shepherd *et al*, 2004).

ZD6474 is an oral, small molecule inhibitor of VEGFR and EGFR tyrosine kinase activity. By targeting these two pathways, ZD6474 has the potential to offer the combined benefits of directly inhibiting tumour cell proliferation and survival like other EGFR inhibitors, as well as inhibiting tumour angiogenesis by inhibiting VEGF activity. In addition, EGFR inhibitors have themselves been shown to exert antiangiogenic effects by reducing the expression of VEGF and other angiogenic factors by tumours (Ciardiello *et al*, 2001), and inhibiting EGFR on tumour endothelium (Bruns *et al*, 2000; Baker *et al*, 2002). Combined blockade of the VEGF and EGFR pathways may therefore provide greater benefit than blockade of either pathway alone. Although clinical experience with combined VEGF/EGFR inhibition is limited, in a recent phase I/II trial of patients with previously treated non-small-cell lung cancer (NSCLC; nonsquamous histology) the combination of erlotinib and bevacizumab showed encouraging antitumour activity (Herbst *et al*, 2005).

## ZD6474 CLINICAL EVALUATION: CONSIDERATIONS IN TRIAL DESIGN

Clinical evaluation of the new generation of molecular-targeted therapies has presented a number of challenges, not least of which is how to design clinical trials that effectively identify the potential of these novel agents. The format of a typical cancer study has developed over many years to evaluate cytotoxic chemotherapy regimens, where tumour regression is the expected outcome for an active agent. In contrast, antiangiogenic agents, like many other types of targeted drugs, are more likely to elicit a cytostatic effect, rather than tumour shrinkage. In addition, while cytotoxic agents are typically used at doses at or near the maximum tolerated dose (MTD), the optimal dose for targeted agents may be at doses far below the MTD. Evaluation of these agents will therefore require refinements in trial design to robustly detect ‘soft’ end points such as stable disease, coupled with the development and validation of improved markers for assessing biological activity.

The principal objective of initial clinical evaluation of ZD6474 was to determine safety and tolerability of this agent, and is discussed below. This followed a conventional format and, as is typical for phase I testing, involved patients with advanced, refractory tumours of various types. The inclusion of a 7-day observation period following initial dosing in these studies also enabled assessment of both single- and multiple-dose effects within the same study. Evidence of tumour responses to ZD6474 therapy seen in the phase I programme and the known activity of EGFR inhibitors in NSCLC prompted a comprehensive phase II evaluation in this tumour type. The main objective of the phase II evaluation of ZD6474 in NSCLC is the assessment of time-to-tumour progression, which is considered the best description of clinical effect of the antiangiogenic agents. Assessment of tumour response is also included as a secondary end point to capture any frank responses to therapy.

Although phase II studies tend not to include a control arm and aim to enrol the minimum number of patients, the use of randomised trials has recently been recommended for screening promising new agents, especially those expected to be cytostatic (Korn *et al*, 2001; Simon *et al*, 2001). In such trials, a one-sided *P*-value of less than 0.2 is considered evidence that the new agent shows sufficient promise to warrant further investigation, that is, a *P*-value of <0.2 would indicate that there was a less than 1 in 5 probability that the observed increase in activity was due to chance alone. A randomised trial design has been adopted for the phase II evaluation of ZD6474 in NSCLC in order to provide more meaningful outcomes than single-arm studies and avoid the necessity for comparison with historical data. The combination studies include a safety run-in phase designed to evaluate the tolerability profile of the combination and to allow early identification of any pharmacokinetic interactions prior to the main randomised phase. Collection of a wealth of additional data is also included in these protocols, including health-related quality of life assessments using the Functional Assessment of Cancer Therapy-Lung (FACT-L) questionnaire and lung cancer subscore. This will provide useful information for determining appropriate outcome end points for future studies.

## PHASE I CLINICAL EVALUATION

**Figure 1:**
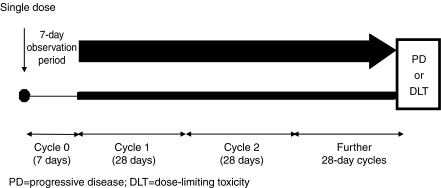
Phase I study design. ZD6474 dosed once-daily p.o. Study A 50–600 mg; study B 100–400 mg.

ZD6474 has been evaluated in two phase I clinical trials of patients with advanced solid tumours refractory to standard therapy (Figure 1). The primary objective of both studies was to evaluate the safety and tolerability of ascending doses of ZD6474. Secondary objectives included assessment of the pharmacokinetic profile of ZD6474, determination of the MTD and preliminary assessment of ZD6474 antitumour activity.

Study A recruited 77 patients from five sites in the USA and Australia (Hurwitz *et al*, 2002). Patients with a variety of solid tumours were included, most having colorectal cancer (CRC) (*n*=23), but only one had NSCLC. Patients received daily oral ZD6474 at doses of 50, 100, 200, 300, 500 or 600 mg in 28-day cycles. Preliminary data from 49 patients shows that good tolerability at doses as high as 500 mg and dosing for >100 days has been achieved. Expanded cohorts of 8–10 patients were also evaluated sequentially at 100 and 300 mg dose levels in order to further characterise the safety, tolerability and biological activity of ZD6474. The incidence of rash and diarrhoea appeared to be dose-related and asymptomatic QTc prolongation was observed in seven patients at varying dose levels. The observation of three CTC grade 3 events in patients receiving 600 mg ZD6474 (two episodes of diarrhoea and one of thrombocytopenia) indicated that this dose level exceeded the MTD and no further dose escalation was performed. ZD6474 exposure (*C*_max_, AUC) following single-dose administration increased with dose. The *t*_1/2_ was long (>100 h) and did not change with increasing dose. The anti-VEGF activity of ZD6474 was suggested by the occurrence of hypertension and delayed dermal wound angiogenesis, although no tumour responses were seen in this study.

**Figure 2:**
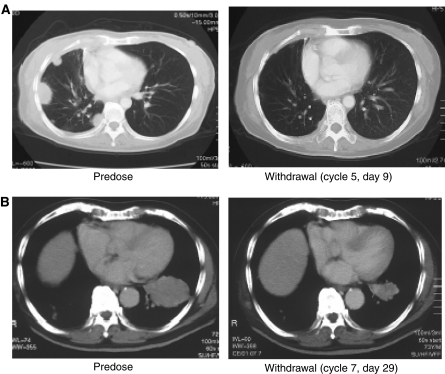
Tumour responses observed in phase I study of ZD6474. (**A**) Female, 50 years, receiving ZD6474 300 → 200 mg day^−1^; (**B**) male, 72 years, receiving ZD6474 200 → 100 mg day^−1^.

Study B recruited 18 patients in Japan, most of whom had NSCLC or CRC (*n*=9 and 4, respectively). Patients received ZD6474 at doses of 100, 200, 300 or 400 mg in 28-day cycles. In this study, ZD6474 therapy was well tolerated at doses of up to 300 mg day^−1^, the most common adverse events being rash, asymptomatic QTc prolongation, diarrhoea, proteinuria and hypertension. Two patients developed a DLT (grade 3 ALT elevation and grade 3 hypertension) at 400 mg day^−1^, which was considered to exceed the MTD. According to RECIST criteria, four of the nine patients with NSCLC exhibited an objective response to ZD6474 therapy. Two of the observed tumour responses are shown in Figure 2, both patients received dose reductions (300–200 and 200–100 mg day^−1^) following an adverse event. It is not known whether these patients had EGFR mutations characteristic of NSCLC patients that are highly responsive to EGFR inhibitors (Lynch *et al*, 2004; Paez *et al*, 2004). In preclinical studies, EGFR mutations known to increase the sensitivity of EGFR to gefitinib and erlotinib also increased sensitivity to ZD6474 (Arao *et al*, 2004; Briggs *et al*, 2005).

In summary, phase I evaluation of ZD6474 has shown this novel agent to be well tolerated at daily oral doses of ⩽300 mg. Observed adverse events were generally mild and readily controlled with dose adjustment or appropriate therapy; all QTc prolongation events were asymptomatic. The pharmacokinetic profile of ZD6474 was compatible with once-daily oral dosing and did not differ between the Western and Japanese studies. This, along with the encouraging tumour response data, has prompted initiation of a comprehensive series of phase II clinical evaluations.

## PHASE II CLINICAL EVALUATION OF ZD6474

Phase II assessment of ZD6474 is now underway in a variety of tumour types in single and combination regimens. ZD6474 doses of 100−300 mg day^−1^ were selected for the phase II studies, as these doses are known to be well tolerated and pharmacokinetic data from phase I evaluation indicate that even the 100 mg dose is expected to yield biologically active systemic concentrations of ZD6474.

## NON-SMALL-CELL LUNG CANCER STUDIES

Although the activity of ZD6474 is being assessed in a range of tumour types, the responses to ZD6474 in patients with NSCLC seen in phase I study B have prompted a series of studies in this tumour type. Current standard therapy for NSCLC has only limited efficacy and although a minority of patients do respond to treatment, the majority of responders will eventually develop progressive disease (Schiller *et al*, 2002). The prognosis for patients with this tumour type therefore remains poor and there is a high unmet need for new therapies.

As with all solid tumours, the role of angiogenesis is well established in the progression of lung cancers, and high microvessel density has been shown to be predictive of tumour progression, metastasis and therefore poor prognosis (Macchiarini *et al*, 1992, 1994; Fontanini *et al*, 1997; Lucchi *et al*, 1997; Meert *et al*, 2002). It is therefore possible that inhibition of VEGF activity and thereby angiogenesis could have significant therapeutic benefits in patients with NSCLC.

## ZD6474 *VS* GEFITINIB IN PREVIOUSLY TREATED NSCLC

**Figure 3:**
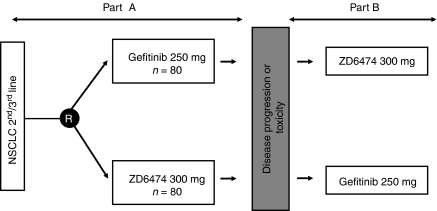
Gefitinib compared with ZD6474; switch-over study design.

Gefitinib is a small molecule inhibitor of the EGFR tyrosine kinase that has demonstrated significant antitumour efficacy in patients with previously treated NSCLC (Fukuoka *et al*, 2003). In order to determine the additional benefit of VEGFR tyrosine kinase inhibition, a comparative study of ZD6474 and gefitinib has been initiated in subjects with locally advanced or metastatic (IIIB/IV) NSCLC after failure of either first-line and/or second-line platinum-based chemotherapy. The crossover design (Figure 3) also allows assessment of the activity of ZD6474 in subjects who have failed treatment with gefitinib. In part A, patients receive daily oral doses of either ZD6474 300 mg or gefitinib 250 mg. The ZD6474 dose selection was based on extrapolations from *in vitro* studies, which demonstrated that the 300 mg dose of ZD6474 could be expected to provide approximately equivalent inhibition of the EGFR tyrosine kinase as gefitinib 250 mg. Treatment continues until withdrawal due to disease progression, toxicity or removal of informed consent. After a washout period of 4 weeks, patients are switched to the alternative treatment (part B), which continues until a withdrawal criterion is reached. In both parts of this study, the dual primary objective is evaluation of time to progression and assessment of safety/tolerability. The initial phase of this study is now complete and the results are anticipated shortly.

## ZD6474 PLUS DOCETAXEL

Docetaxel is currently a standard chemotherapy agent for previously treated patients with NSCLC. In randomised phase III studies, the overall response rate has ranged from 6 to 9%, with a median progression-free survival of 8.5−12.6 weeks (Shepherd *et al*, 2000; Fossella *et al*, 2000; Hanna *et al*, 2004). Docetaxel has also been found to act synergistically with VEGF pathway inhibitors in preclinical studies (Sweeney *et al*, 2001).

**Figure 4:**
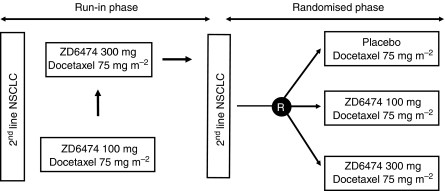
ZD6474/docetaxel combination study design.

The potential for improved therapeutic efficacy in combination with ZD6474 is being investigated in a two-part randomised, double-blind, placebo-controlled trial (Figure 4). Patients with locally advanced or metastatic NSCLC after failure of first-line platinum-based chemotherapy were recruited and initially received docetaxel (75 mg m^−2^ i.v. infusion every 21 days) plus daily oral ZD6474 (100 or 300 mg) in the open-label safety run-in phase. In the subsequent randomised phase, a docetaxel/placebo group was included. The primary objective of this study is to compare progression-free survival, as determined by objective tumour assessments (revised RECIST).

A randomised study, rather than a single-arm protocol, was performed as the activity of docetaxel observed to date in second-line NSCLC trials has been highly variable, making comparison with historical data difficult. In addition, the inclusion of a control arm allows the tolerability profile of the combination arms to be rigorously placed into context with that observed for docetaxel alone.

**Figure 5:**
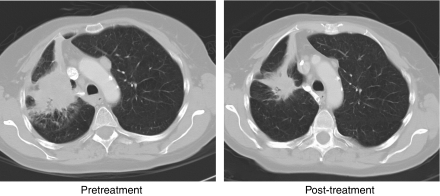
Tumour response following two 21-day cycles of ZD6474 (300 mg day^−1^) plus docetaxel (75 mg m^−2^ every 21 days) therapy.

Preliminary data from the run-in phase of this study have demonstrated that the combination of ZD6474 and docetaxel does not significantly increase the toxicity or exposure of either agent (Heymach *et al*, 2004). The combination was generally well tolerated and adverse events were manageable. Approximately half of the patients exhibited stable disease ⩾12 weeks and two partial responses were seen in the ZD6474 300 mg cohort (Figure 5). Overall median time to disease progression was 15.1 and 19.8 weeks in the ZD6474 100 and 300 mg cohorts, respectively. There was a higher incidence of rash in the 300 mg cohort, possibly due in part to a higher degree of EGFR inhibition. These data therefore support progression to the double-blind, randomised phase, which has now completed accrual.

## ZD6474 PLUS CARBOPLATIN AND PACLITAXEL

**Figure 6:**
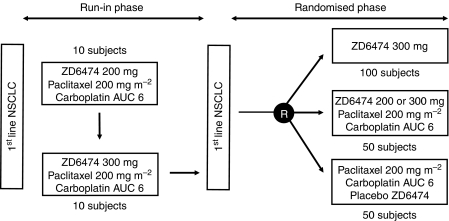
Design of carboplatin/paclitaxel/ZD6474 combination study in first-line NSCLC patients.

ZD6474 is being evaluated in an ongoing randomised, double-blind phase II study, alone and in combination with carboplatin/paclitaxel (CP), as first-line treatment for patients with locally advanced or metastatic (IIIB-IV) NSCLC (Figure 6). A safety run-in phase was conducted to establish the appropriate dose of ZD6474 to be administered in combination with CP, and comprised 21-day treatment cycles in which 10 subjects per cohort received daily oral doses of ZD6474 (1st cohort, 200 mg; 2nd cohort, 300 mg) in combination with CP (carboplatin, target AUC_ss_=6 mg ml^−1^ min; paclitaxel, 200 mg m^−2^ i.v.). In the randomised phase, the inclusion of a control arm will allow the tolerability profile of the ZD6474/CP combination arm to accurately compare with that of CP only.

Preliminary data from the run-in phase of this study show that the ZD6474/CP combination is generally well tolerated (Johnson *et al*, 2005). Partial responses have been observed in seven out of 18 patients and stable disease ⩾12 weeks in a further two patients. Preliminary pharmacokinetic data indicate that mean steady-state plasma levels of ZD6474 after 200 mg dosing are comparable when given alone (684 ng ml^−1^±272) or in combination with CP (568 ng ml^−1^±206). These preliminary results have supported progression to the randomised phase, which is currently ongoing.

It is worth noting that two previous phase III studies of CP combined with either gefitinib or erlotinib did not demonstrate improved survival compared with CP alone (Herbst *et al*, 2004a, 2004b). When CP was combined with the VEGF inhibitor bevacizumab, however, promising activity was seen in terms of overall response rate and time to progression (Johnson *et al*, 2004). A phase III trial of this regimen (ECOG 4599) has now completed accrual. Given these results, it will be of particular interest to determine whether a dual VEGF/EGFR inhibitor will increase the effectiveness of CP chemotherapy for first-line NSCLC.

## CLINICAL EVALUATION OF ZD6474 IN OTHER TUMOUR TYPES

ZD6474 as monotherapy has been evaluated in a phase II study of 46 patients with previously treated metastatic breast cancer. In this study, patients received once-daily ZD6474 therapy at a dose of 100 or 300 mg (Miller *et al*, 2004). ZD6474 therapy was generally well tolerated, with mild diarrhoea and rash being the most commonly reported adverse events. There were no objective responses seen in this study. Phase I evaluation of bevacizumab in patients with advanced cancers, including breast cancer, also showed no responses (Gordon *et al*, 2001). A subsequent phase I/II dose escalation study in previously treated metastatic breast cancer patients has shown more promising results, with a confirmed response rate of 6.7% (Cobleigh *et al*, 2003).

It is possible that the lack of clinical benefit in the ZD6474 study may be due to the advanced, previously treated nature of the patient group. It is also possible that early-stage tumours have a greater reliance on VEGF as the principal proangiogenic factor, while in advanced tumours there may be a degree of redundancy, with angiogenesis being governed by a wider range of proangiogenic factors (Relf *et al*, 1997; Pavlakovic *et al*, 2001; Kerbel, 2004). Also, while EGF is an important mediator of normal mammary gland development and neoplastic transformation, the value of inhibiting EGF functioning in the treatment of breast cancer remains uncertain. Further research will elucidate whether certain tumour types are more or less susceptible to anti-VEGF/EGF strategies, and breast cancer may prove to be a tumour type that is difficult to treat with this approach.

Other studies of ZD6474 include small-cell lung cancer (SCLC) and multiple myeloma (MM). The ongoing SCLC study is recruiting patients who have experienced a complete or partial response to induction chemotherapy±radiation therapy. The primary objective is assessment of time to progression. In addition, a correlation between outcome or response and tumour VEGFR expression and microvascular density will be assessed. As MM is a disease in which angiogenesis is believed to be a relevant target for therapy, a phase II trial of ZD6474 100 mg p.o. daily in patients with relapsed MM has been conducted. Although ZD6474 therapy was well tolerated in this population, the primary efficacy end point, objective response as assessed by reduction in M protein, was not achieved (Kovacs *et al*, 2004).

## CONCLUSIONS

ZD6474 has the potential to exert both direct and indirect antitumour effects, via inhibition of VEGFR-2 and EGFR tyrosine kinase activity, respectively. Early clinical evaluation of this novel agent has demonstrated a promising efficacy and safety profile and the comprehensive series of ongoing phase II studies will clarify the value of this approach in the treatment of solid tumours. Our knowledge of tumour biology suggests that targeting multiple pathways in tumour growth and development will be critical, although early clinical experience indicates that there is still much to be learned about how to optimally combine these targeted agents with one another and with chemotherapy. Careful patient selection may be necessary in order to ensure targeted therapy is administered to those most likely to gain clinical benefit from this novel therapeutic approach. The identification of surrogate markers of treatment efficacy will be key to attaining this goal and is the subject of much ongoing research.
